# Supplementary material for: Versatile Double Bandgap Photonic Crystals of High Color Saturation
Source: Nanomaterials (Basel). 2023 Sep 24;13(19):2632. doi: 10.3390/nano13192632 (PMC10574206; doi:10.3390/nano13192632)
Supplement: Supplementary file 1 [file nanomaterials-13-02632-s001.zip › SI-nanomaterials.pdf]

Versatile Double-Bandgap Photonic Crystals of High Color Saturation

Hao Jiang [1], Gang Li [2], Luying Si[1], Minghui Guo[2], Huiru Ma [3,4*], Wei Luo [2,3*] and Jianguo Guan [1,3]

1 State Key Laboratory of Advanced Technology for Materials Synthesis and Processing, International School of Materials Science and Engineering, Wuhan University of Technology, Wuhan 430070, China; jh1998@whut.edu.cn (H.J.); siluying@whut.edu.cn (L.S.); guanjg@whut.edu.cn (J.G.)
2 School of Materials Science and Engineering, Wuhan University of Technology, Wuhan 430070, China; chemligang2010@whut.edu.cn (G.L.); gmh1232000@163.com (H.G.)
3 Wuhan Institute of Photochemistry and Technology, 7 North Bingang Road, Wuhan 430083, P. R. China
4 School of Chemistry, Chemical Engineering and Life Science, Wuhan University of Technology, Wuhan 430070, China
* Correspondence: mahr@whut.edu.cn (H.M.); rowell@whut.edu.cn (W.L.)

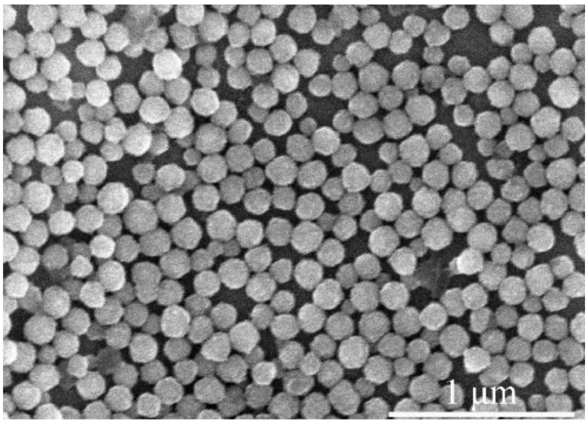

Figure S1. SEM image of Fe_3O_4@PVP superparamagnetic particles with a diameter of 150 nm.

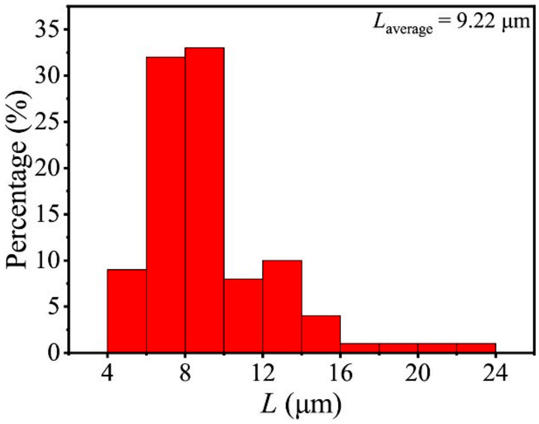

Figure S2. The length distribution diagram of green PNCs.

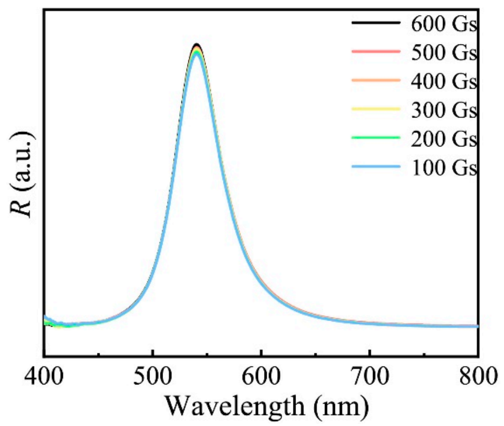

Figure S3. Reflection spectra of green PNCs under different magnetic field.

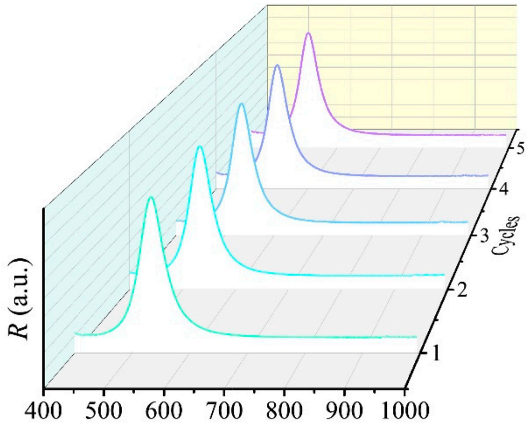

Figure S4. The cycling stability of PNCs under repeatedly applied magnetic field.

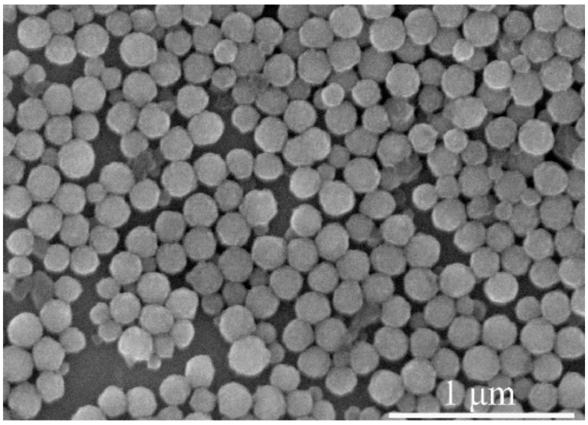

Figure S5. SEM image of Fe_3O_4@PVP superparamagnetic particles with a diameter of 180 nm.

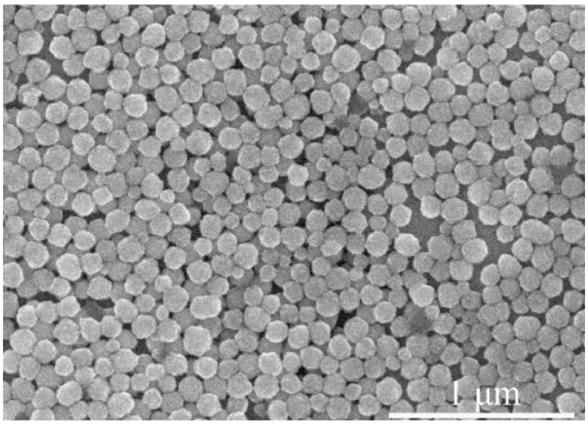

Figure S6. SEM image of Fe_3O_4@PVP superparamagnetic particles with a diameter of 120 nm.

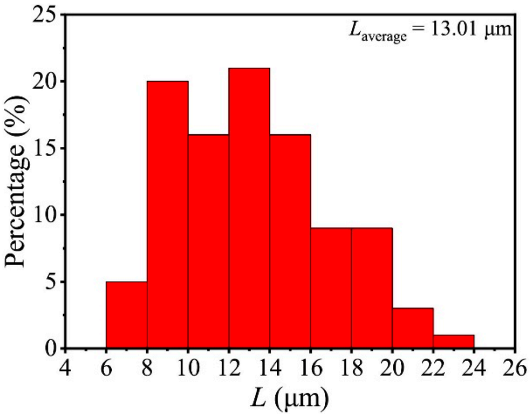

Figure S7. The length distribution diagram of red PNCs.

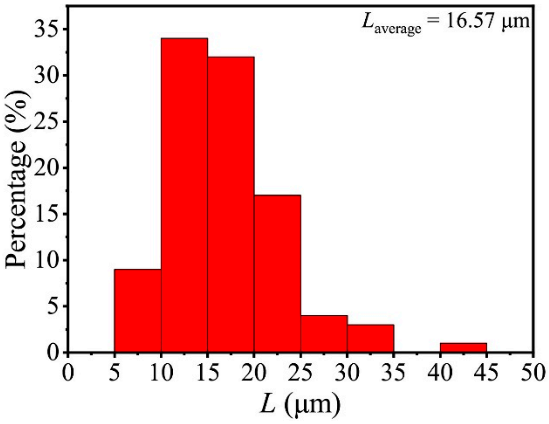

Figure S8. The length distribution diagram of blue PNCs.

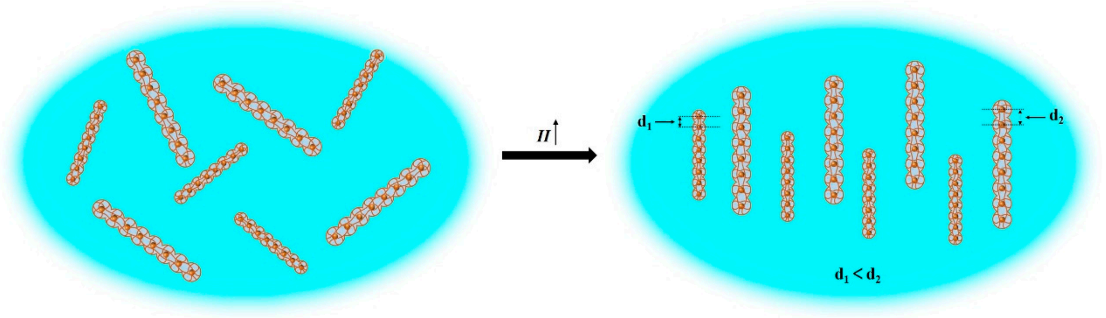

Figure S9. Magnetic induced orientation of two type of PNCs with different lattice distance ($d_1 < d_2$) in an external magnetic field.

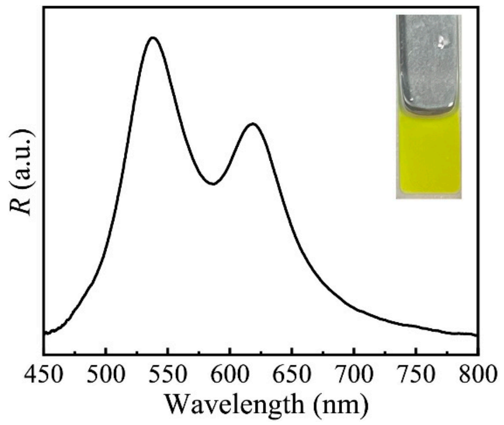

Figure S10. The reflection spectrum and digital photo of mixed PNCs (more green PNCs).

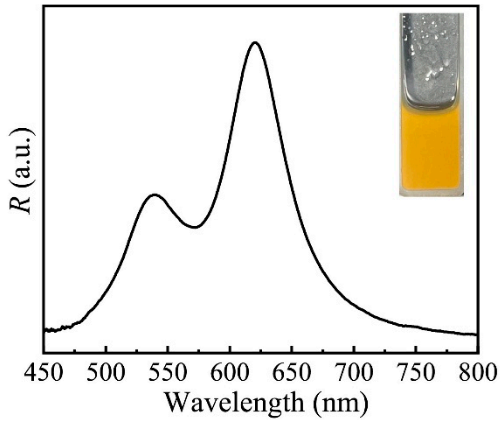

Figure S11. The reflection spectrum and digital photo of mixed PNCs (more red PNCs).
